# Effect of Soil Geomechanical Properties and Geo-Environmental Factors on Landslide Predisposition at Mount Oku, Cameroon

**DOI:** 10.3390/ijerph17186795

**Published:** 2020-09-17

**Authors:** Wamba Danny Love Djukem, Anika Braun, Armand Sylvain Ludovic Wouatong, Christian Guedjeo, Katrin Dohmen, Pierre Wotchoko, Tomas Manuel Fernandez-Steeger, Hans-Balder Havenith

**Affiliations:** 1 Department of Earth Sciences, Faculty of Sciences, University of Dschang, Dschang P.O. Box 67, Cameroon; wdjukem@gmail.com (W.D.L.D.); armand.wouatong@univ-dschang.org (A.S.L.W.); suhguedjeo@gmail.com (C.G.); 2 Department of Engineering Geology, Institute of Applied Geosciences, Faculty VI Planning Building Environment, Technische Universität Berlin, 10587 Berlin, Germany; anika.braun@tu-berlin.de (A.B.); k.dohmen@tu-berlin.de (K.D.); fernandez-steeger@tu-berlin.de (T.M.F.-S.); 3 HTTC Bambili, University of Bamenda, Bamenda P.O. Box 39, Cameroon; pierrewotchoko69@gmail.com; 4 Geology Department-B18, Georisk and Environment, Faculty of Sciences, Liege University, B-4000 Liege, Belgium

**Keywords:** soil geomechanical properties, geo-environmental factors, pearson correlation coefficient, statistical index information value method, fuzzy membership, receiver operator characteristic (ROC) curve, landslide susceptibility, disaster prevention

## Abstract

In this work, we explored a novel approach to integrate both geo-environmental and soil geomechanical parameters in a landslide susceptibility model. A total of 179 shallow to deep landslides were identified using Google Earth images and field observations. Moreover, soil geomechanical properties of 11 representative soil samples were analyzed. The relationship between soil properties was evaluated using the Pearson correlation coefficient and geotechnical diagrams. Membership values were assigned to each soil property class, using the fuzzy membership method. The information value method allowed computing the weight value of geo-environmental factor classes. From the soil geomechanical membership values and the geo-environmental factor weights, three landslide predisposition models were produced, two separate models and one combined model. The results of the soil testing allowed classifying the soils in the study area as highly plastic clays, with high water content, swelling, and shrinkage potential. Some geo-environmental factor classes revealed their landslide prediction ability by displaying high weight values. While the model with only soil properties tended to underrate unstable and stable areas, the model combining soil properties and geo-environmental factors allowed a more precise identification of stability conditions. The geo-environmental factors model and the model combining geo-environmental factors and soil properties displayed predictive powers of 80 and 93%, respectively. It can be concluded that the spatial analysis of soil geomechanical properties can play a major role in the detection of landslide prone areas, which is of great interest for site selection and planning with respect to sustainable development at Mount Oku.

## 1. Introduction

Soil geomechanical properties have long been investigated to characterize the behavior of soils from a small scale (grain or particle) to a landslide scale. Additionally, soil geomechanics overlap with parts of geotechnical engineering and provide useful information about soil mechanical behavior. Previous studies in the field of soil mechanical behavior have shown that landslides occur suddenly. A landslide is a complex geological process of denudation-erosion, which involves the displacement of soil or rock along a slope under the influence of gravity [1,2]. However, the study of rock and soil characteristics, faults, lineaments, and seismic events allows detecting warning signs, including small displacements of parts of the slope, tension cracking, and reactivation of spring lines [3,4,5,6]. Furthermore, soil geomechanical properties, such as water content, porosity, grain sizes, plasticity index, methylene blue value (MBV), soil angle of internal friction, and cohesion, can play a significant role when trying to understand and predict slope soil failure mechanisms [3,7,8,9].

**Figure 1:**
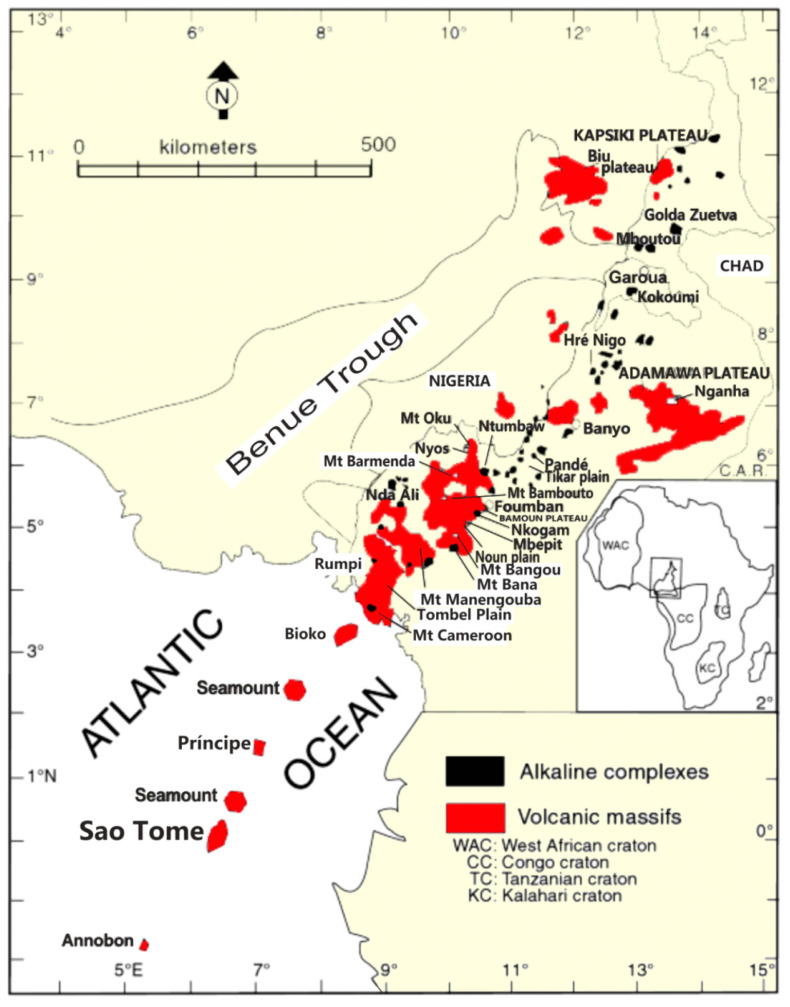
A map of the study area showing locations of alkaline complexes of plutonic origin (black) and volcanic massifs (red) of the Cameroon Volcanic Line [27,28].

Land exploitation, combined with heavy rainfall and slope steepness, frequently cause various types of natural hazards in tropical, mountainous regions [10,11]. Floods and landslides are among those destructive natural disasters frequently experienced in these regions. Landslide hazards are frequent in Cameroon, especially along the Cameroon Volcanic Line (CVL), which is a 1600-km-long mega-shear zone in Central Africa (Figure 1). Mount Oku, culminating at an altitude of 3011 m, is situated on the continental part of the CVL. It is one of the great volcanoes of the CVL, with Mounts Bamenda, Cameroon, Manengouba, and Bambouto [12,13,14]. This area presents a humid, tropical, highland climate and steep hill slopes with deeply incised valleys. The study area encompasses about 20 villages with a population density of 162.3 inhabitants/km^2^, according to the 2005 national institute of statistics census. The Mount Oku area plays a significant role in the northwest region in terms of infrastructure, grazing, and agriculture—therefore, it is important to better constrain the conditions that could lead to landslide disasters in that area and to provide indications allowing to prevent them [10,15,16].

Landslide-susceptibility investigation consists of computing the spatial distribution of areas prone to landslide [17,18,19]. This involves investigating the relationship between various parameters, ranging from lithology, topography, climate, land use, hydrogeologic and soil geotechnical properties, and landslide occurrence. For seismically active and volcanic regions, as the one investigated here, also related factors have to be taken into consideration (especially the first ones are studied more in detail in a new paper under preparation).

Literature on the understanding and prevention of landslides through susceptibility assessment in Mount Oku area is still scant, as also recently noticed in the western branch of the East African Rift region by [20,21]. Landslide studies conducted in Cameroon can be classified into four approaches. In the first approach, the authors studied landslide-predisposing factors including morphological, geological, hydrological, and geographical properties [15,22,23]. In this approach, no geotechnical characteristics of soils were taken into account, and landslide-conditioning factors were simplified. In the second approach, relief and geology were identified as causative factors, amplified by triggers, such as rainfall, small earthquakes, human activities, and mechanical erosion [16,24]. Nevertheless, in these models, landslide factors were considered separately, without integrating them. In the third approach, applied by [25,26] at Magha, West Cameroon, soil mineralogical, geochemical, and geotechnical characteristics were identified as major landslide controls, neglecting the role of morphological and other spatially distributed factors. In the fourth approach, Ref. [8] emphasized slope soils’ geotechnical properties along the Bamenda escarpment but did not include them in their landslide-predisposition model. Thus, it can be observed that there is a tendency to use either morphological data layers, which are easily available from digital elevation models and satellite imagery, or only scattered data that require detailed field investigations. Moreover, these previous studies at Mount Oku revealed that the qualitative approach, including landslide spatial and temporal distribution (inventory) and a heuristic approach (based on the researcher experience), is actually the most used compared to quantitative methods, which are more reliable.

In spite of the relevance of soil geomechanical properties in slope failure investigations, they are rarely integrated in landslide-susceptibility models. Consequently, in this study, we investigated the effects of soil geomechanical properties on landslide-susceptibility models in the Mount Oku area (Figure 1).

Soil geomechanical properties and geo-environmental factors were mapped, classified, and weighted based on their landslide-causative influences, using the statistical index information value [29,30,31,32] and the fuzzy membership method [33,34,35,36]. Then, we generated three different landslide-susceptibility models, one soil geomechanical model, one geo-environmental model, and one combined model in order to investigate the relevance of geomechanical factors in landslide-susceptibility mapping.

## 2. Study Area

### 2.1. Climate and Geological Setting

**Figure 2:**
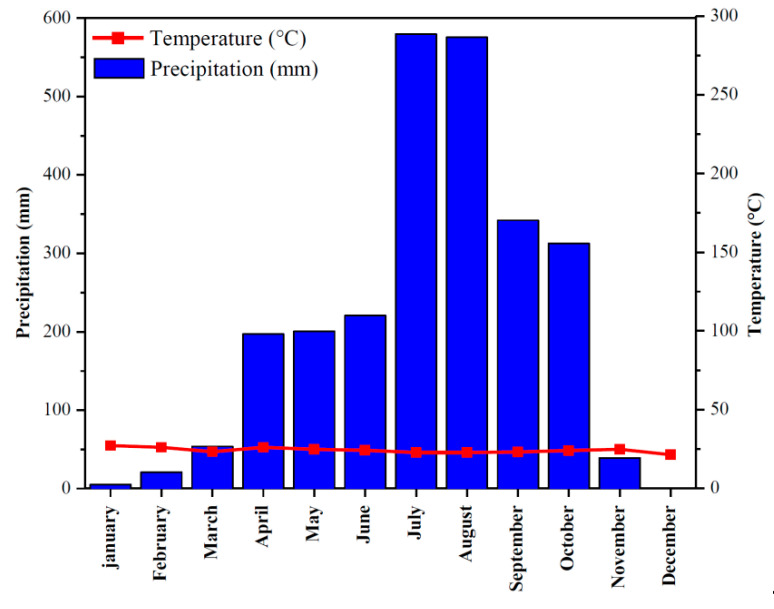
Average annual rainfall and temperature distribution for Bamenda. Source: Northwest regional service for meteorology. These average monthly precipitation/temperature data were recorded between 2005–2010.

The study area is located in a humid, tropical, highland climate with two seasons. The rainy season begins in April and ends in November (eight months), with a total precipitation of 2467 mm. The dry season ranges from December–March (four months), with a total precipitation of 80 mm. A transition from dry to rainy season can be observed in March, with a slight increase of precipitation above the temperature curve (Figure 2). The total annual precipitation is 2546.5 mm. The maximum rainfall is observed during the month of July (579.6 mm) and the minimum during the month of December (0 mm), as shown in Figure 2. The temperature data shows that January (27.2 °C), February (26 °C), and April (26 °C) are hot, while the months of July and August are cold (22.7 and 22.8 °C, respectively). The mean annual temperature is 24.5 °C at an elevation of approximately 1200 m.

The Oku massif volcano, where the study area is located, dates from an Oligocene volcanism (about 33.9 ± 0.1 to 23.03 ± 0.05 million years) and belongs to the Western Highlands [13,14,37,38,39,40]. Mount Oku is located within the Cameroon Volcanic Line, characterized by an alignment of oceanic and continental volcanic massifs and plutonic complexes. The Oku massif volcano is a complex stratovolcano with a diameter of approximately 90 km and a height of 3011 m and is located near the central part of the CVL continental sector [41]. This complex stratovolcano shelters four major stratovolcanoes, which are Mount Oku, Mount Babanki, Nyos, and Nkambe. References [13,42] demonstrated that the continental part of the CVL consists of both plutonic and volcanic massifs, while the oceanic part is made only of volcanic massifs. Volcanic rocks show mostly basic lavas, forming rocks such as basalt and hawaiite. Felsic lavas form rocks such as trachyte, phonolite, and rhyolite with very few intermediate rocks (mugearite and benmoreite). However, plutonic rocks present a complete series of gabbro-diorite-monzonite-syenite-granite type. Geochemically, trace elements in basalts are nearly similar in both oceanic and continental sectors of the CVL, implying a related upper mantle source. Mount Oku eruptive products vary from basanite and alkali basalt through hawaiite and mugearite to trachyte-rhyolite, with high-level intrusions of pyroclastic material. The basement rocks upon which the lavas were erupted include granites, migmatites, and biotite diorites [41].

Superficial formations originate from the physical and chemical weathering of these bedrocks either in place or during their transportation over long distances, after which they are deposited in lowlands. Mount Oku soils are mostly made of sand, silt, and clay in different proportions.

### 2.2. Landslides in the Study Area

**Figure 3:**
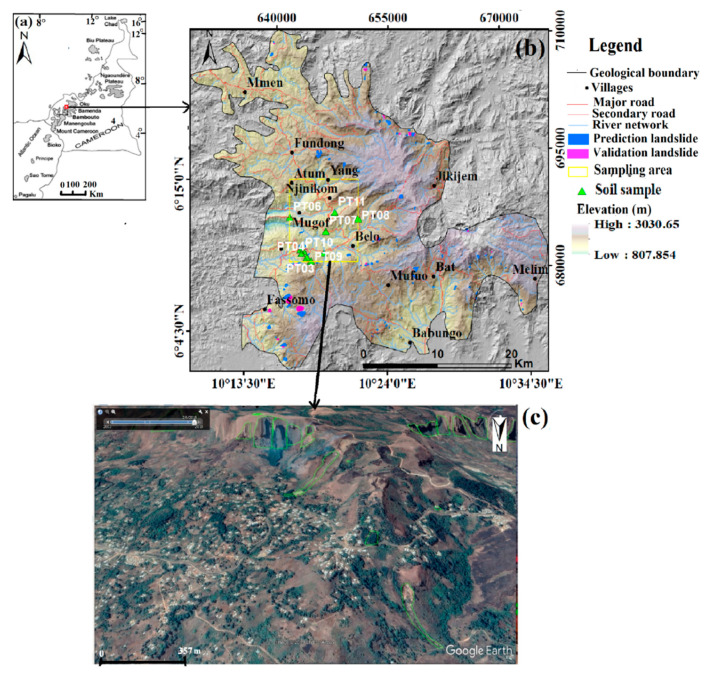
(**a**) Mount Oku location on the Cameroon Volcanic Line. The study area situated on the central part is symbolized by the red square (modified from [42]); (**b**) an enlargement of this area with the 12-m TANDEM X-derived elevation as base map, showing landslides (yellow rectangle); (**c**) Google Earth image showing landslides (green polygons) observed between 2009–2018.

This investigation was carried out on the western flank of Mount Oku (Figure 3a) between latitude 6°2′20″ N to 6°25′23″ N and longitude 10°11′39″ E to 10°35′46″ E, especially on four rock types, namely, basalts, trachy-rhyolites, rhyolites, and migmatites. These rocks cover approximately 955 km^2^ (6 302 739 12 × 12 m pixels). Landslides cover an area of about 10 km^2^, representing almost 1% of the study area. Old and recent landslide locations were recorded in the field using a GPS and mapped with the use of the MapSource application (Garmin Ltd., Schaffhausen, Switzerland), Google Earth (Google LLC, Mountain View, CA, USA), and ArcGIS software (ESRI Inc., Redlands, CA, USA). Seventy-five percent of landslides (42,147 pixels) were introduced in the prediction models and 25% (14,067 pixels) of the most recent landslides in the validation model.

The study area limit refers to the geological boundary, as denoted on Figure 3b. The weathering of the rock formations present in the study area led to the formation of gentle to very steep slope clayey soils based on grain size distribution. The diverse geo-environmental factors prevailing in the study area and soil properties make these slopes prone to slope movements.

**Figure 4:**
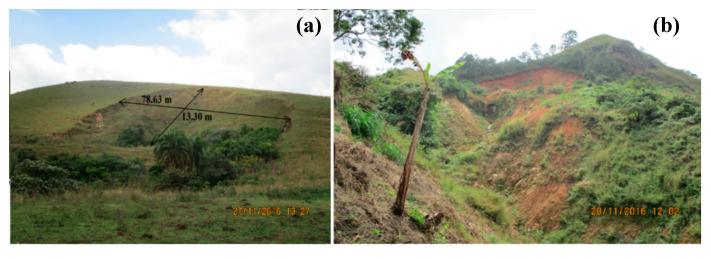
Deep-seated translational landslides observed at Mbingo: (**a**) Translational, (**b**) translational failure transformed into debris flow.

A total of 179 small- to large-size, shallow to deep slides, debris flows, and complex slides from liquefaction or flow processes were identified using Google Earth images and field observations, following mainly the classification system of [43], as shown in Figure 3c. Shallow landslides have their slip surfaces not deeper than 3 m, while deep-seated landslides display rupture surfaces below 3 m, based on the classification of [44]. A major landslide with a maximum depth of failure surface of about 13.30 m was observed at Mbingo (Figure 4a). Additionally, Figure 4b presents a recent complex landslide, which presented a translational mechanism at failure, but was transformed into a debris flow that travelled approximately 200 m downstream.

## 3. Materials and Methods

### 3.1. Soil Sampling and Laboratory Testing

**Figure 5:**
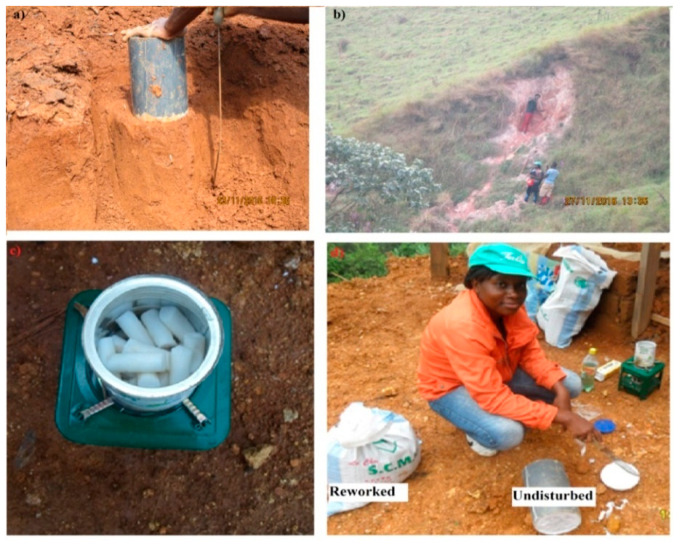
Sampling: (**a**) Undisturbed soil sampling method; (**b**) sampling in the major landslide found at Mbingo; (**c**) stove used to melt candles; (**d**) reworked and undisturbed samples ready for laboratory analyses.

Undisturbed and disturbed soil samples were collected on flat and hilly surfaces, above escarpments, on anthropogenic cuttings, and in scars at depths between the ground surface and the basal slip plane, as recommended by [45]. Representative soil samples were taken at 0.25 m to 7.3 m depth, on failure and nonfailure sites, from soils developed on each rock type, flat and sloping areas, in forested and nonforested areas, and close to major rivers and roads. Undisturbed samples were collected using cylinders of 18 cm in diameter and 25 cm in height, as illustrated in Figure 5. These cylinders were placed on the soil surface and inserted with manual pressure. When the cylinder was full, the soil column was cut at the base. Melted candle wax was then poured on both sides of the cylinder to prevent any evaporation of water from the sample.

Geomechanical properties of these soils were determined through physico-mechanical laboratory experiments (particle and bulk densities, moisture content, grain size distribution, Atterberg limits, methylene blue, and direct shear tests). Five of these samples were analyzed at the Sol Solution Afrique Centrale laboratory, Yaoundé, Cameroon. In this laboratory, the tests were performed following the procedures from the American Society of Testing Material (ASTM) and French Standardization Association (AFNOR) standards, since they are appropriate for fine-grained soils analysis, as noticed by [46,47].

The six remaining samples were analyzed at the Engineering Geology Department of the Technische Universität Berlin, Germany, following the standards proposed by [48], which is a manual for the laboratory and field testing of soils for civil engineers, soil engineers, and technicians. Results of these tests are presented in Section 4.1.

Shear test results allowed obtaining the shear strength parameters friction angle ($\phi_{UU}$) and cohesion $(c_{UU}$). These parameters are linked by the Mohr–Coulomb strength criterion, Equation (1), which is the most common strength criterion applied to soils as mentioned by [49,50].

(1) $$\tau_{ff}={\;\sigma }_{ff}\cdot \tan(\phi_{\mathrm{UU}})+{\;c\;}_{\mathrm{UU}}\Rightarrow {\;c}_{\mathrm{UU}}=\tau_{ff}-\sigma_{ff}\cdot \tan(\phi_{\mathrm{UU}})$$

where τ_ff_ is the shear strength on the failure plane at failure, σ_ff_ is the applied normal stress on the failure plane at failure, Φ_UU_ is the angle of internal friction or friction angle under undrained unconsolidated conditions, and c_UU_ is the intrinsic cohesion under undrained unconsolidated conditions.

### 3.2. Analysis of Soil Geomechanical Properties

The degree of association between the soil geomechanical properties pairwise was evaluated using the Pearson correlation coefficient. These coefficients and the corresponding matrix table were generated using the Microsoft Excel software. The Pearson correlation coefficient varies between −1 and +1; the nearer the coefficient is to one of these limits, the stronger the relationship. The signs indicate if the compared properties are increasing or decreasing together when it is positive, or if one is increasing while the other decreases for a negative sign [51,52].

### 3.3. Mapping of Soil Property-Based Landslide Susceptibility

Soil property maps were realized using the laboratory test results, of which mean values were first assigned to the corresponding geological features, using Microsoft Excel and Esri ArcGIS 10.1 software. Spatial interpolation of geomechanical properties was done using the fuzzy logic or fuzzy membership approach earlier used by [33,53,54,55]. With this method we could avoid having corresponding weight values identical to those of the geology units, as would be the case using the information value method. Soil geomechanical properties were first mapped by attributing their mean value to the corresponding geology units, as they result from their weathering. Then, these soil properties maps were converted into fuzzy subsets with values between 0–1. Assigning membership functions to each data set was carried out according to the relation between soil geomechanical properties’ behavior and slope failure processes. Therefore, we converted these soil geomechanical properties into fuzzy subsets following analyses of the Pearson correlation coefficients, literature on soil properties’ influence on landslides, and textural and Casagrande diagrams, noted above. The assignment of fuzzy membership values was performed using the Fuzzy membership option of the Spatial Analyst tools in ArcGIS 10.1. More specifically, the “Linear” fuzzy functions of this tool were used. The “Linear” fuzzy function calculates membership based on the linear behavior of the input raster. It assigns membership values between 0 for the minimum and a membership of 1 for the maximum. These normalized geomechanical properties were then overlaid using the Fuzzy Gamma operator to identify landslide-susceptible areas. The gamma operation is defined in terms of the fuzzy algebraic product and the fuzzy algebraic sum by:

(2) $$\mu \mathrm{combination}\;={\left(\mathrm{fuzzy}\;\mathrm{algebraic}\;\mathrm{sum}\right)}^{\gamma }\times \;{\left(\mathrm{fuzzy}\;\mathrm{algebraic}\;\mathrm{product}\right)}^{1-\gamma }$$

where γ values are chosen between 0–1. The γ is equal to 1 when the combination is the same as the fuzzy algebraic sum, where the combined geomechanical property raster is more important than any single of them; and γ is equal to 0 when the combination is identical to the fuzzy algebraic product, in which the combined geomechanical property raster is less important than any single of them. A suitable γ value ensures a good compromise between the “increasing” tendencies of the fuzzy algebraic sum and the “decreasing” effects of the fuzzy algebraic product [14,55,56].

### 3.4. Bivariate Statistical Analysis of Geo-Environmental Factor-Based Landslide Susceptibility

Next, geo-environmental properties were mapped, classified, and reclassified into various classes, while landslide maps for prediction and validation were reclassified into landslides and no landslides areas [57,58]. The influence of each landslide factor class was determined by assigning coefficients (weight), quantifying their degree of influence on landslide occurrence based on the bivariate statistical information value method. This method provided good results in many landslide-susceptibility mapping studies, such as [23,59,60]. In this work, it was used to determine the weight or influence of each landslide factor class by dividing the density of landslides in each factor class by the density of landslides in the entire map. The density of landslides within each class of a factor is equal to the ratio of the number of pixels of landslides within class i and the number of pixels of class i. Landslide density within the study area is equal to the ratio of the total number of landslide pixels within the whole area over the number of pixels of the whole study area. Then, the statistical index information values obtained were rasterized using the “lookup tool” of the spatial analyst extension and summed up using the raster calculator. The weight of each class of a factor is given by Equation (3) below [30,31,32]:

(3) $$W_{i}=ln\frac{Density\;of\;landslides\;within\;each\;class\;\mathrm{of}\;a\;factor}{Density\;of\;landslides\;within\;the\;study\;area}=ln\left(\frac{{\mathrm{Npix}}_{\left(Si\right)}}{{\mathrm{Npix}}_{\left(Ni\right)}}/\frac{{\Sigma \mathrm{Npix}}_{\left(Si\right)}}{{\Sigma \mathrm{Npix}}_{\left(Ni\right)}}\right)$$

With

*Wi* = weight of each class;
Npix (*Si*) = number of pixels of landslide within class i;
Npix (*Ni*) = number of pixels of class i;
ΣNpix (*Si*) = number of pixels of landslide within the whole study area;
ΣNpix (*Ni*) = number of pixels of the whole study area; and
*ln* = natural logarithm used to take care of the large weights variation

The following landslide-predisposing and causative factors were used for landslide-susceptibility mapping with 12 × 12 m cell size, based on the geo-environmental conditions prevailing in the study area and data availability. They are:

(a) Elevation, slope angle, aspect, and curvature maps prepared from the TerraSAR-X add-on for Digital Elevation Measurements (TanDEM-X DEM) standard product 0.4 arcsecond with 12.37 m resolution at the equator, kindly provided for this research by the German Aerospace Center (Deutsches Zentrum für Luft- und Raumfahrt, commonly known as DLR);
(b) A geological map that was digitized from the existing Wum-Banyo 1:500,000 geological map (sheet number 4 32 NE—040 and 41) translated and digitized by Peronne and published in 1969, by the Office for Scientific Research in Overseas Territories (ORSTOM), Directorate of Mines and Geology; and other recent geological maps from articles such as those of [13,14], combined with field and laboratory works;
(c) Distances from the main roads that were acquired by digitizing the road network in Google Earth and computing the distance from the road network using the Euclidean distance tool of ArcGIS 10.5, using the 1:50,000 topographic map Nkambe 1b (sheet number NB-32-XVII-1b) drawn and published in 1969 by the French National Geographical Institute (NGI);
(d) Distances from major rivers that were computed with the Euclidean distance tool around rivers that were digitized from the Nkambe 1b topographic map; and
(e) A land cover map that was prepared from the LANDSAT 8 satellite image recorded by LANDSAT on 14 January 2019, by applying a supervised classification in ArcMap and field surveys for more accuracy. Bands 4 and 5 were used to calculate the normalized difference vegetation index (NDVI) with the raster calculator tool of the ArcGIS 10.5 Spatial Analyst tools.

The classification of elevation, slope angle, and curvature maps was executed using the quantile classification method. In this method, the class intervals are adapted to the distribution of the data, so they are more evenly distributed among the classes. Regarding the distance parameters (distance to roads and rivers), several classes were created at closer distances to the targeted roads and rivers with a cutoff where no more effects are expected. These classes are 0–200 m, 200–400 m, 400–600 m, 600–800 m, 800–1000 m, 1000–1200 m, 1200–1400 m, and ≥1400 m. Moreover, for categorical factors, such as lithology and aspect, all the classes of the nominal scale were preserved.

### 3.5. Landslide-Susceptibility Model Generation and Evaluation

Three different landslide-susceptibility models were generated and normalized. The first was obtained by adding the resulting weights of geo-environmental factors (lithology, elevation, slope angle and aspect, land use, curvature, and proximity to rivers and roads). The second model was obtained by summing up the fuzzy memberships of soil geomechanical properties (porosities, particle and bulk density, water content, grain sizes, Atterberg limits, methylene blue value, friction angle, and cohesion). In the third model, both geo-environmental factors and geomechanical properties were merged using the raster calculator of the ArcGIS 10.5 Spatial Analyst tools.

Finally, the predictive power of these landslide-susceptibility models was assessed using the receiver operator characteristics (ROC) curves and the area under the ROC curve (AUC), as also used for several landslide investigations [20,61,62,63,64,65]. The first step for the ROC curve implementation is to overlay the validation landslide map (landslides and nonlandslide areas) with the landslide-susceptibility models that were reclassified into unstable and stable pixels and divided into 100 equal intervals. The landslide-susceptibility index value of 0.5 was used as cutoff to discriminate between stable and unstable areas. Four possible predictive situations can, therefore, be observed:

(a) Areas of observed landslides, which have been predicted by the model to be unstable, also called true positive (TP);
(b) Stable areas of no landslides, classified by the model as stable, also called true negative (TN);
(c) Areas without landslides but predicted by the model to be unstable, also designated as false positive (FP); and
(d) Areas with landslides but predicted by the model to be stable, also named as false negative (FN).

Table 1 displays a matrix table, which is frequently used to tabulate the model and experiment predictive results and calculate the sensitivity and 1-specificity, which are later plotted on a graph to obtain the ROC curve. The true positive rate (TPR) or sensitivity, which corresponds to the percentage of landslides or true positive events, correctly predicted as unstable pixels, is plotted on the *Y*-axis. The false positive rates (FPR) or 1-specificity, which is the proportion of nonlandslide areas or truly negative events, correctly predicted as stable pixels, is plotted on the *X*-axis [61,63]. TPR and FPR are computed using the following formulas:

(4) $$\mathrm{TPR}=\frac{\mathrm{TN}}{\mathrm{TN}+\mathrm{FP}}$$

(5) $$\mathrm{FPR}=\frac{\mathrm{TP}}{\mathrm{TP}+\mathrm{FN}}$$

Finally, the predictive power of these models was evaluated by comparing the AUC values of the three landslide-susceptibility models, as recommended by [61]. The nearer the curve is to the upper-left corner of the ROC graph or above the diagonal line (corresponding to AUC = 0.5), the better the model. Additionally, a perfect model would have an AUC value of 1.

## 4. Results and Discussion

### 4.1. Soil Geomechanical Properties and Their Influence on Slope Stability

The bivariate correlation of soil geomechanical properties was analyzed by computing the Pearson correlation coefficient and using the textural and Casagrande diagrams. Geomechanical properties determined at the Sol Solution Afrique Centrale Laboratory, Yaoundé Cameroon (PT01, PT02, PT03, PT04, and PT05 samples) and the Engineering Geology Department of the Technische Universität Berlin, Germany (PT06, PT07, PT08, PT09, PT10, and PT11 samples) are presented in Table 2, with PT meaning a pit from which soils samples were removed.

These results, obtained in both laboratories, are homogeneous with minor variations among each group of values. Soil sampling depths in this study fluctuated between 0.25–7.3 m, corresponding to the average depth of landslide slip surfaces recorded at the western flank of Mount Oku. The degree of linear relationship between them pairwise was determined by calculating the Pearson correlation coefficient. The Pearson correlation matrix displayed in Table 3 shows that these properties are all linearly interconnected. In this table, there are one strong negative correlation ≤ 0.7, 16 moderate negative correlations ≤ 0.4, 10 negligible/weak negative correlations (between −0.40), 14–negligible/weak positive correlations (between 0–0.4), 10 moderate positive correlations > 0.4, and two strong positive correlations > 7, based on the classification proposed by [52]. It is also noted that relationships between bulk and particle densities, sand, silt and clay contents, plasticity index, friction angle, and cohesion are stronger with many properties compared to porosity, water content, and methylene blue value, as shown by their high correlation coefficients.

Additionally, bulk and particle densities, porosity, fine particle content (clay and silt), cohesion, and friction angle have positive correlation coefficient signs, supposing that they increase or decrease together. Contrarily, plasticity index, methylene blue values, water content, and sand percentages are supposed to vary in the opposite direction with cohesion, regarding their correlation coefficient signs, which are negative. When they are increasing, cohesion is supposed to decrease and the liquefaction potential increases, leading to landslides. Additionally, sandy soils exhibit no cohesion [66], but they display a high liquefaction potential. Furthermore, Ref. [67] in their study concerning the influence of ants on soil and water losses in eastern Spain, they found a reduction in soil bulk density and an increase in macropore flow in ant-affected soils, which makes them prone to landslides. This supports our observations of densities being interrelated with soil grain sizes, water content, porosity, consistency limits, absorption capacity, cohesion, and friction angle. Therefore, it can be concluded that landslides at Mount Oku are closely influenced by their soil geomechanical properties.

Although the linear correlation is strong between most of these properties, there could also be a positive or negative nonlinear, monotonic relationship [67,68]. Therefore, other correlation approaches are needed to confirm these soil properties correlations before introducing them in landslide-causative factors systems. In addition to the Pearson correlation coefficient, many authors investigated the relationships among soil properties, as presented below.

Moreover, soil samples from Mount Oku exhibited water amounts that exceed their plasticity index values, as already noticed above. In this state, water exerts pressure on soil pores, decreasing the friction just as the shear strength responsible for the material stability is also reduced. This points out a possible initiation mechanism of slope instabilities that can trigger landslides, if they are combined with other factors, such as rainfall and steepness of the slope [69,70,71]. Additionally, water causes a decrease in shear strength either by reducing the apparent soil cohesion or by creating or extending cracks, which represent potential slip surfaces when moistened. This is directly related to intense or long-lasting rainfall events, as also shown by [72].

**Figure 6:**
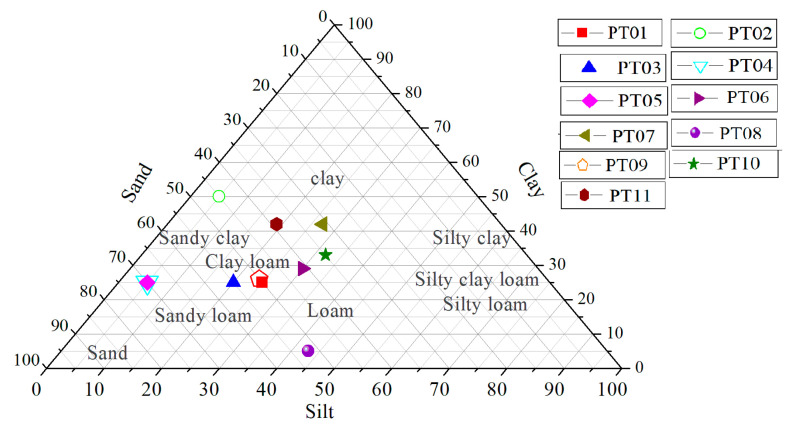
Texture triangle showing proportions of clay, sand, and silt particles of samples at nonfailures (PT01, PT02, PT01, PT02, PT06, PT07, PT08, PT09, PT10, and PT11) and failures, or landslides, sites (PT03, PT04, and PT05); modified from [74,75].

The proportions of clay, sand, and silt particles presented in Table 2 were plotted on the texture diagram, as shown in Figure 6. It can be seen that nonfailure sites correspond to PT01, PT02, PT06, PT07, PT08, PT09, PT10, and PT11 samples and failure or landslide sites are locations of PT03, PT04, and PT05 samples.

The samples from landslide sites are rather in the sandy regime, while the samples from the nonlandslide sites have higher fine particle contents, since failure phenomena depend greatly on the grain size, as concluded by [73] after their investigation of the pore-pressure generation and movement of rainfall-induced landslides in laboratory flume tests.

**Figure 7:**
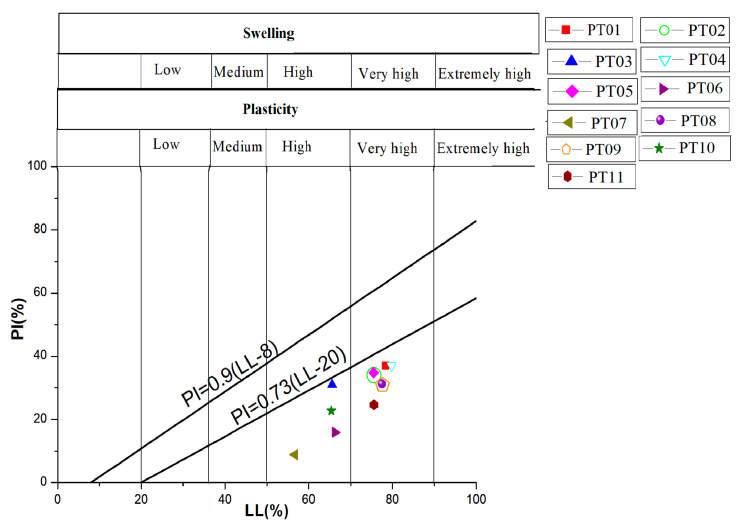
Casagrande’s diagram showing plasticity and swelling capacity. The samples show high to very high plasticity and swelling potential (modified from [47]).

Furthermore, liquid limit and plasticity index values of Mount Oku soils reported on the Casagrande diagram describe a cloud of points with low dispersion (Figure 7). This diagram shows that the samples PT03, PT06, PT07, and PT10 are highly plastic with a great swelling potential. PT01, PT02, PT04, PT05, PT08, PT09, and PT11 are very plastic with excessive swelling potential.

In view of all this, soil samples were finally classified as highly plastic clays with a high to very high swelling behavior, showing that they can experience swelling and significant shrinkage in the presence or absence of water [76,77]. Swelling and shrinkage alternation can create weaknesses in these soil textures, easing their failure. Similar observations have been made by [9,77] in their investigations concerning slope shear and strength deterioration through drying–wetting succession and influence of synthetic wick fibers on clayey soils’ behavior, respectively.

It, therefore, follows that the interdependence of soil geomechanical properties is directly related to landslide occurrences. This is supported by the statistical correlation of these properties with landslides as displayed below.

### 4.2. Spatial Analysis of Soil Geomechanical Properties with the Fuzzy Membership Approach

The average values of soil geomechanical properties were computed for each rock type, as can be seen in Table 4. These soils on basalt, trachy-rhyolite, and migmatite displayed almost similar average values of geomechanical properties. Particle and bulk densities varied between 2.59–2.66 g/cm^3^ and 1.15–1.58 g/cm^3^, respectively. Water content and porosity varied from 39.3–44.1% and 43–45%, respectively. The differences in sand, silt, and clay content among soils are less than 25%. Soils on basalt and trachy-rhyolite display the most similar geomechanical properties. Soil on migmatite displays the highest bulk and particle densities, porosity, silt, clay, friction angle, and cohesion mean values. Moreover, they show the lowest water content, sand, and plasticity limit mean values. Rhyolites were assumed, based on literature, to have a bulk density of 26.5 g/cm^3^, cohesion of 1000 kPa, and 46° as friction angle [78]. They display no porosity, individual grain sizes, plasticity limits, water content, and methylene blue values.

Many authors have shown that the increasing or decreasing tendency of soil geomechanical properties can create weaknesses in these soil textures, easing their failure or sliding [7,69,74]. In other words, some of these geomechanical properties should be decreasing to make the site more susceptible to landslides. This is the case for bulk and particle densities, internal angle of friction, and cohesion (Table 5). The fuzzy membership values of bulk density classes vary from 0–0.99, while those of particle density, cohesion, and friction angle fluctuate between 0–1.

However, in other cases, rising of soil geomechanical properties increases the probability of landslide occurrence. This is the case for porosity, water content, MBV, PI, sand, and clay content (Table 6). The fuzzy membership values of porosity, water content, MBV, PI, sand, and clay contents classes vary between 0–1.

### 4.3. Bivariate Correlations of Geo-Environmental Factors with Landslides

**Figure 8:**
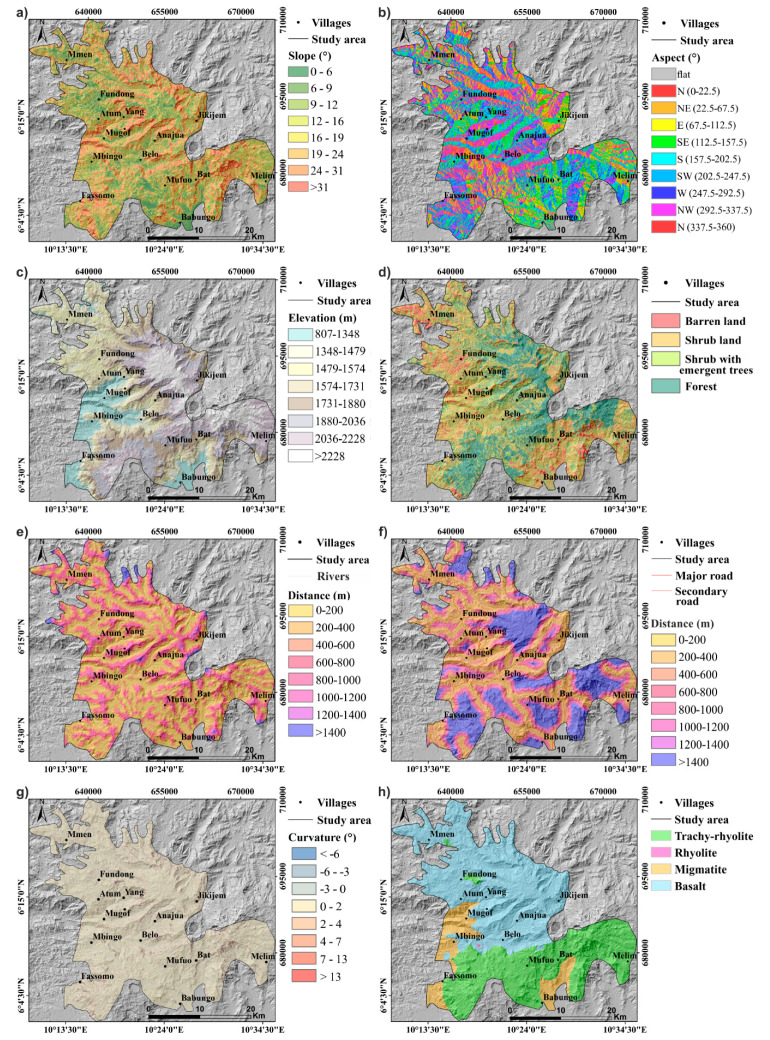
Geo-environmental factors maps reclassified into various classes with hill shade as base map: (**a**) Slope angle, (**b**) slope aspect, (**c**) elevation, (**d**) land cover, (**e**) distance to rivers, (**f**) distance to roads, (**g**) curvature, and (**h**) lithology.

Slope angle and aspect, land use, elevation, distances from the main road, lithology, and distances from the major stream maps are presented in Figure 8.

(a) Slope Angle

**Figure 9:**
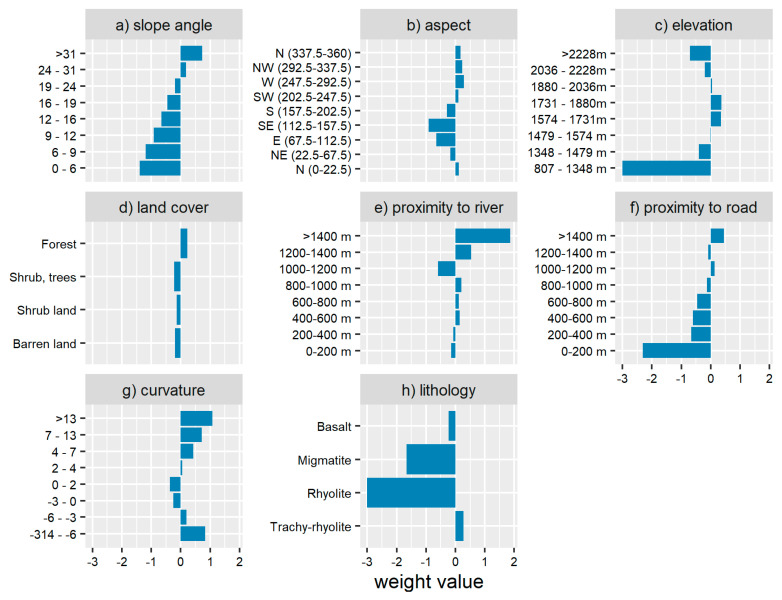
Geo-environmental factors with their corresponding classes and weights: Negative weight means the class parameter has less influence on landslide events and positive weight values show that the class parameter has a significant effect on landslides in this area.

Slope angle, which is the inclined surface of the land, is among the most determining factors of landslide types and velocities [18]. In this area, very gentle slopes (0–15°) and gentle slopes (15–25°) occupy the largest surface of this area, but they have less influence on landslide occurrences, while slope angles ranging from 24–31° and >31° (Figure 8a), respectively, present the highest positive weights of 0.18 and 0.73, correspondingly (Table 7a and Figure 9). Their highest positive weights can be due to interactions between slope steepness and other factors, such as shear stress produced by the material weight and road and river cuttings, which modify the natural slopes and destabilize the block material [10,11].

(b) Slope Aspect

Slope aspect (Figure 8b) is the direction toward which the surface of the slope faces. At Mount Oku, most slopes are directed toward north (0–22.5°), southwest, west, northwest, and north (337.5–360°), as shown in Table 7 and Figure 9, and are more influential with, respectively, 0.05, 0.12, 0.26, 0.23, and 0.20 as weights, while others are negative. Indeed, meteorological events in Mount Oku are likely to be more intense mainly on northern-, southwestern-, western-, and northwestern-oriented slopes. This can be attributed to the fact that slope aspect, vegetation cover, soil water retention, rainfall depending on the wind direction, and sunshine intensity are interrelated, as shown by [79] in their investigation of the effect of elevation and aspect on wind temperature and humidity. The low intensity of sunshine, intensified soil moisture, and weathering on these slopes is leading to landslides events [11,80,81].

(c) Elevation

The elevation classes (Figure 8c) between 1574–1731 m and 1731–1880 m display the highest weight values of 0.24 and 0.25 each, as shown in Table 7a and Figure 9. Elevation is an indirect landslide factor as it has an influence on other factors, such as rainfall, temperature, soil development, and vegetation [82,83].

(d) Land Cover

Land cover of the study area (Figure 8d) has been categorized into four classes, namely, barren land, shrub land, shrub with emergent trees, and forest. The forest areas have the highest weight values of 0.22 (Figure 9 and Table 7a). The positive weight displayed by the forest cover can be explained by the fact that in Mount Oku the population used to stabilize landslide scars by planting trees, such as eucalyptus, which later grew into forest. Moreover, in this area, the natural forest plays a role as a stabilizer for steep slopes through promotion of infiltration and drainage. Similar conclusions were made by [81] concerning landslide-susceptibility mapping with information value and logistic regression methods in the Bailongjiang watershed in China. Similarly, Ref. [72] established that the amount of water entering a slope depends on several geomorphological factors, anthropogenic activities, and atmospheric conditions including vegetation type, drainage, soil type, and rock structure.

(e) Proximity to Major Rivers

On the western flank of Mount Oku, most landslides are found at distances between 400–600 m, 600–800 m, 800–1000 m, 1200–1400 m, and >1400 m from rivers (Figure 8e). These represent the highest information values of 0.14, 0.12, 0.20, 0.54, and 1.87, respectively, while others are negative (Figure 9 and Table 7b). Deep, incised river channels can modify the natural slope at the toe of mountains through water erosion. It also influences groundwater level fluctuation, which is related to intensive soil wetting and drying phenomenon that lead to slope instability [81]. Here, however, there is no clear relationship between proximity to rivers and landslide occurrence and the classes with high weight values seem relatively random. Especially the high weight values in the classes >1200 m could be related to the small class size, an effect that could be considered to be an artifact.

(f) Proximity to Main Roads

Major landslide scars are found at 1000–1200 m and >1400 m from the roads, with weight values of 0.13 and 0.45, respectively, and highly negative values closer to the roads (Table 7b, Figure 8f and Figure 9). Usually, the presence of roads is believed to trigger landslides through undercutting of slopes. As a result of an increase in stress on the back of the slope, due to changes in topography and decrease of load on the toe, tension cracks may develop [59,80]. Here, however, the high weight values observed in the classes far away from the roads, at 1000–1200 m and >1400 m, and negative values close to roads seem, again, quite random.

This effect can be explained by the fact that these landslides close to rivers and roads are of rather small sizes. Therefore, even if they are many landslides, they could be regarded as insignificant at the scale of the study area.

(g) Curvature

Curvature allows identifying the shape of the slope. It plays an important role in erosion and deposition processes by defining the convergence and divergence of water flow. Curvature classes ranging from −314 to −6°, −6 to −3°, 2 to 4°, 4 to 7°, 7 to 13°, and >13° have the highest information values of 0.82, 0.20, 0.05, 0.42, 0.73, and 1.08, each (Table 7b, Figure 8g and Figure 9). As presented by [84,85], the landslide movement direction together with driving and resisting stresses along the failure slope are influenced by curvature, since it controls the speed and convergence or divergence of landslide-displaced material and water flowing down the slope.

(h) Lithology

The study area is covered by volcanic rocks, precisely basalt (highly weathered with thick, residual soil), rhyolite (slightly weathered, no residual soil, and indicated by an arrow on the map), trachy-rhyolite (moderately weathered with very steep slopes), and migmatites (highly weathered with moderately steep slopes). Trachy-rhyolite covers 37%, while basalt covers 54% of the study area (Figure 8h). Trachy-rhyolite displays the highest information values of 0.27. Migmatite, basalt, and rhyolite present negative information values of −1.66, −0.23, and −3, respectively (Figure 9 and Table 7b). This is probably due to variations in thickness, steepness of slopes, and strength of soils developed from the weathering of these rocks. This has also been noted by [18,86] in their research on soft rock mass-weathering effect on slope stability and debris flow susceptibility assessment in Subao river valley, respectively.

Geo-environmental factor classes, with their corresponding area percentages and weights, calculated using the information value technique, are presented in Table 7a,b and Figure 9. Some classes of these features have the highest positive weight values, demonstrating their higher landslide prediction ability in this zone, as also noticed by [23] in their work on landslide-susceptibility mapping on the Bamenda mountain. The sum of each geo-environmental factor-positive class weights show that lithology is the dominant landslide factor in the western flank of Mount Oku, followed, respectively, by slope angle, curvature, land use, aspect, proximity to road, proximity to rivers, and elevation. However, it was difficult to determine which geomechanical properties were the most important, since their spatial distribution was identical to rock type.

### 4.4. Landslide-Susceptibility Model Results and Discussion

**Figure 10:**
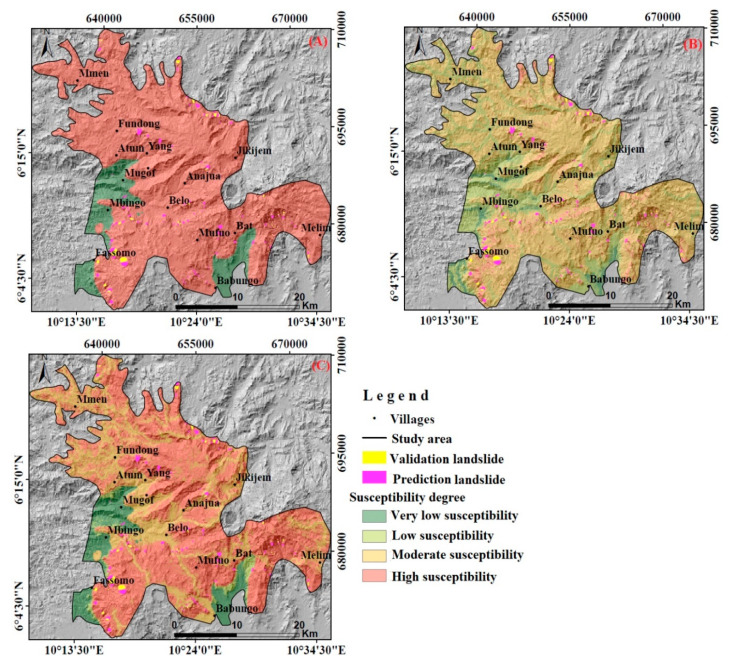
The landslide-susceptibility models classified into four classes: (**A**) Soil properties; (**B**) geo-environmental factors; (**C**) soil properties merged with geo-environmental factors.

**Figure 11:**
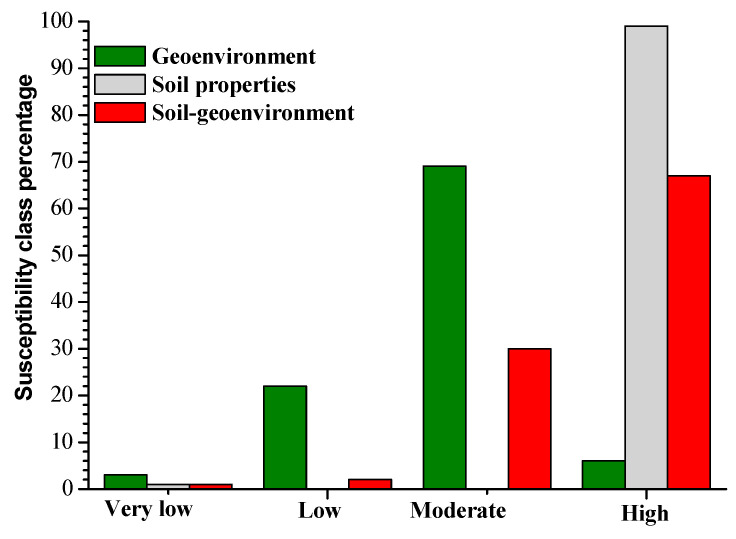
Variation of the landslide-susceptibility models class percentages: Geo-environmental factors in dark green; soil properties in light grey; soil properties merged with geo-environmental factors in red color.

In order to thoroughly evaluate the significance of geo-environmental factors and soil geomechanical properties, three landslide-susceptibility models were established with the first one merging only soil properties, geo-environmental factors for the second, and the last one combining all geo-environmental factors and soil properties. These models present areas of identified landslides and areas with similar predisposing conditions, where landslides have not yet been experienced, as also noticed by [18,19], among others. The resulting landslide-susceptibility map indexes have been normalized and classified using the equal interval method, so the results could easily be compared with each other. The landslide-susceptibility maps were classified into four susceptibility areas: High, moderate, low, and very low (Figure 10). The very-low-susceptibility class ranged between 0–0.25, the low between 0.25–0.50, the moderate from 0.5–0.75, and the high one from 0.75–1. For the model with only geo-environmental factors, 6% of the study area was highly susceptible, 69% was moderately susceptible, 22% was lowly susceptible, and 3% was very lowly susceptible (Figure 10 and Figure 11). The landslide-susceptibility model results presented here are justified based on established methods and existing literature on geomechanical properties, relationships among the geomechanical properties, and geo-environmental and geomechanical factors in landslide-susceptibility assessment.

The model based only on soil properties indicates 99% to be highly susceptible, 0% moderately susceptibility, 0% lowly susceptible, and 1% to be very lowly susceptible. The combined model shows most of the study area, 67%, to be highly susceptible, 30% moderately susceptible, 2% lowly susceptible, and 1% to be very lowly susceptible (Figure 11). Basalt and trachyte are highly weathered with thick, residual, clayey soils that are highly plastic and, hence, sensitive to the variations of water content. Soils on trachyte and basalt may rapidly pass from liquid to plastic or solid state, predisposing this area to landslides, when combined with steep slopes. This makes these soils more susceptible to landslides than shallow, residual soils developed on migmatites, which is reflected by the model. Furthermore, soils on migmatites display relatively gentle slopes, moderately weathered with shallower, residual soil, low porosity, and high cohesion values, making them less susceptible to landslides. Besides this, Ref. [86,87] stated that when the water content values vary between 30 and 40%, the degree of swelling is medium, as it was the case in this work with soils on migmatite. This soil also presents a high cohesion value, which means that the grains are strongly cemented.

A slight variation was observed between the susceptibility class percentages of the model with only geo-environmental factors and those of the model merging geo-environmental factors and soil properties. This is mostly because the weights of geomechanical properties obtained with the fuzzy membership method are small (from 0–0.74). These models allow observing some variations between the susceptibility class percentages. A distinctive maximum of the high susceptibility class percentage was observed in the model combining only soil geomechanical properties, while the minimum was displayed by the geo-environmental factors model. Moreover, the lowest percentages of very low and low susceptibilities were observed in the soil properties and the soil-geo-environment models. In other words, the susceptibility class percentages of the model with only geo-environmental factors and the one with only soil properties presented either the lowest or the highest susceptibility class percentages (Figure 11). The combined model of soil properties and geo-environmental factors tended to be the most reasonable and stable. When soil properties or geo-environmental factors were used alone, the resulting landslide-susceptibility model tended to misjudge the susceptibility degree. The soil, which is the material concerned by slides, allows a more precise examination of landslide predisposition conditions when combined to other landslide-predisposing factors, such as geo-environmental factors in this case. The soil of an area classified with low landslide predisposition regarding geo-environmental conditions can hide some characteristics that situate it at the stability limit or out. These characteristics can include tension cracking, spring lines mostly due to high swelling, and shrinkage capacities, as previously stated by [3,4,5,6,9].

One limitation of the soil geomechanical properties model is clearly the lack of spatial variability of the three different soil units that follow the spatial distribution of the geological units. This explains also the large size of the high susceptibility class that is also affecting the result of the combined models. The combined model, however, showed a much better spatial distinction of areas of different landslide susceptibility. Moreover, also the geo-environmental factors model showed a very large medium- and high-susceptibility class. Such large areas of high landslide susceptibility may lead to high prediction rates, but they are not very useful in terms of efficient targeting of measures to reduce landslide damage. A more adequate method for the classification of the landslide susceptibility could help to improve the effectiveness of the resulting landslide-susceptibility maps.

**Figure 12:**
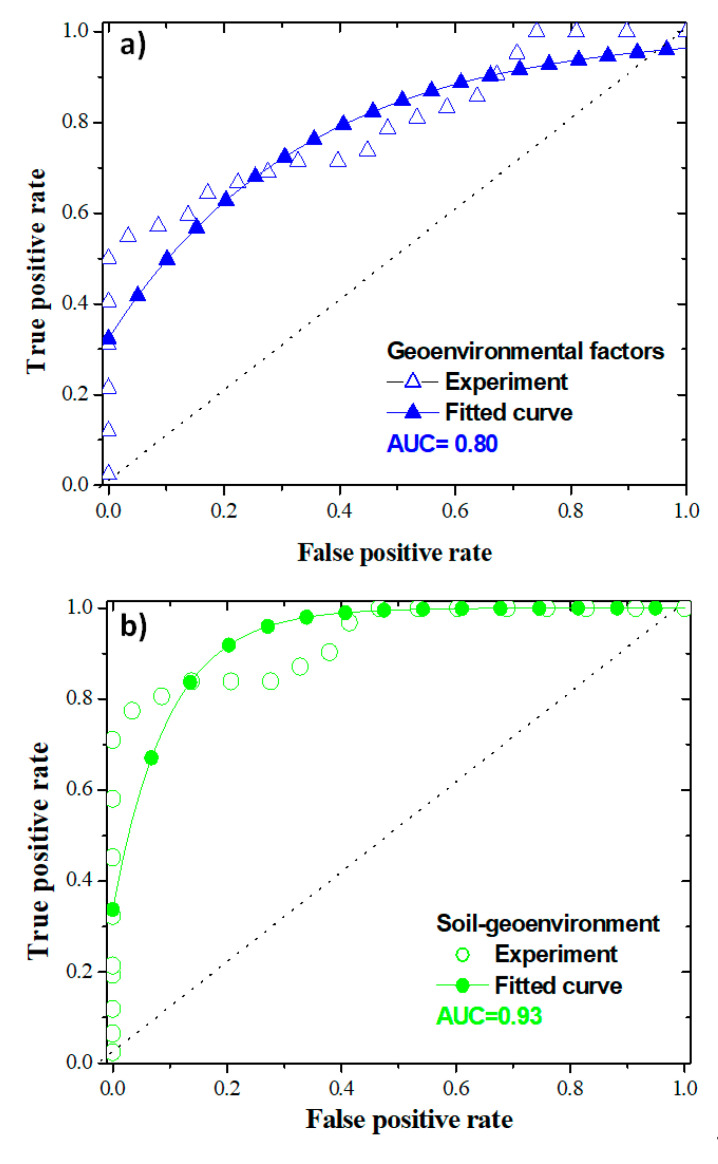
ROC curves for: (**a**) Model based on geo-environmental factors only, (**b**) model based on soil geomechanical and geo-environmental factors.

The prediction accuracy of the landslide-susceptibility models was evaluated using the receiver operator characteristic (ROC) curve and the area under the curve (AUC). Comparison of ROC curves and corresponding AUC values are presented in Figure 12. The ROC curve of the model combining only soil properties was not computed because of its low spatial variability (this map could not be classified into more than three pixel categories). The ROC curves of the geo-environmental and combined soil-geo-environmental models display for each, an experimental and a model-fitting curves. The experimental curves of these models describe an exponential profile according to equation (Equation (6)), generated in the Origin 6.1 software (OriginLab Corporation, Northampton, Massachusetts, USA) using the “Nonlinear Curve Fit” option and presented below:

(6) ROC(t)=TPR0+ A1−FPRt1

The characteristics of Equation (6): true positive rate (*TPR*_0_), coefficient (*A*_1_,) false positive rate (FPR) and the threshold *t*_1_ are given in Table 8.

The curves of the models with only geo-environmental factors and the combined model present AUC values of 0.80 and 0.93, respectively (Figure 12). These AUC values traduce the high efficiency of these models in landslide-susceptibility prediction and show that the combined model with soil properties and geo-environmental factors is the most efficient in the identification of future landslide events of the western flank of Mount Oku. Thus, it can be noted that soil properties increase the predictive power of the model with only geo-environmental factors. Therefore, to take efficient measures in order to reduce landslide damages, geo-environmental factors and geomechanical properties should be combined in landslide-susceptibility assessment.

## 5. Conclusions

In this study we intended to propose a novel approach to integrate both geo-environmental and soil geomechanical parameters in a landslide-susceptibility model at Mount Oku (NW-Cameroon). To achieve this goal, the Pearson’s correlation coefficient, soil textural triangle, and Casagrande’s diagram were used to evaluate relationships between soil geomechanical properties, stability limits, and swelling capacity of soil samples from the study area. Furthermore, fuzzy membership values were assigned to the mean values of soil properties, in order to quantify their effect on landslide occurrence. The information value method was used to quantify the influences of geo-environmental factors on landslide occurrence through assigned weights. Soil properties, geo-environmental feature maps, and landslide-susceptibility models were computed with the obtained weights and fuzzy membership values. On the basis of these results, the following conclusions were drawn:

(a) Mount Oku soil geomechanical properties are strongly correlated and can be merged with other landslide factors.
(b) High water content and related properties could exert pressure on Mount Oku soil pores, decreasing the internal friction angle and the shear strength responsible for the material stability.
(c) Atterberg limits and grain size distribution allowed classifying soils as highly plastic clays with a high to very high swelling and shrinkage potential, which favored landslide occurrences.
(d) The increasing or decreasing tendency of each soil geomechanical property that favors landslide occurrences were mapped using the fuzzy membership approach.
(e) Statistical correlation of the selected geo-environmental factors and landslide pixels revealed the higher prediction ability of some geo-environmental factor classes.
(f) When only considering soil properties, the resulting landslide-susceptibility model tended to underrate unstable and stable areas. When soil properties were combined with geo-environmental factors, a more precise identification of stability conditions was possible.
(g) The model with geo-environmental factors had an AUC value of 0.80, which means that this model is suitable for landslide prediction in this area. Meanwhile, the model with soil properties and geo-environmental factors displayed an AUC of 0.93, which traduces its excellent predictive capacity. From this result it can be concluded that soil geomechanical properties play an important role in the identification of landslide-prone areas. Soil geomechanical properties of some areas classified as very low or low landslide susceptibility, with respect to the prevailing geo-environmental conditions, can comprise some physico-mechanical effects that revealed their relative instability.

## Figures and Tables

**Table 1:** Matrix table for the comparison of landslide-susceptibility models (test results) and validation landslides (true condition).

Test	Stability Degree	
**Test result**	Unstable	Stable
Negative	TN	FN
Positive	FP	TP

TN: true negative, FN: false negative, FP: false positive, TP: true positive.

**Table 2:** Geomechanical properties of soil samples.

Sample	D	$\rho_{s}\;$	$\rho_{b}\;$	W	n	Sand	Silt	Clay	PI	**MBV**	$\phi_{UU}$	**CUU**	**Soil on**
PT01	0.7	2.498	1.394	44.3	44.2	50	25	25	36.9	7.27	12	2	Basalt
PT02	0.45	2.422	1.492	35.7	38.4	45	5	50	34	1.47	13.6	5	Basalt
PT03	0.55	2.379	1.544	46	35.1	55	20	25	31	2.67	11.3	20	Trachyte
PT04	0.55	2.569	1.365	42.4	46.87	70	5	25	37	1.6	9.1	20	Trachyte
PT05	0.25	2.584	1.403	48.1	45.7	70	5	25	34.8	2.53	9.6	7	Basalt
PT06	1.6	2.9	1.584	39.29	45.39	31	27	42	15.9	1.54	31.5	47.7	Migmatite
PT07	2.5	2.755	1.508	44.54	45.25	41	30	29	8.8	1.09	29.6	46.5	Basalt
PT08	2.1	2.848	1.794	37	36.99	39	19	42	31.2	1.12	41.2	15	Basalt
PT09	3.7	2.848	1.468	44	48.46	35	32	33	30.9	0.5	29.9	59.6	Trachyte
PT10	1.1	2.814	1.466	24.05	47.89	48	23	25	22.7	0.4	30.7	58.6	Basalt
PT11	7.3	2.74	1.452	54.62	47.02	52	43	5	24.6	0.24	28.6	43.9	Basalt

D: sampling depth (in m), ρ_s_ and ρ_b_: particle and bulk densities (in g/cm^3^), W: water content (in wt.%), n: porosity (in %), PI: plasticity index (in %), MBV: methylene blue value (in g/100g), ϕ_UU_: friction angle (in °), C_UU_: cohesion (in kilo Pascal).

**Table 3:** Correlation matrix visualizing Pearson’s coefficients for geomechanical properties.

Parameters	$\rho_{b}$	$\rho_{s}$	W	n	Sand	Silt	Clay	PI	MBV	$\phi_{UU}$	**C_UU_**
$\rho_{b}$	1										
$\rho_{s}$	0.402	1									
**W**	−0.220	−0.259	1								
**n**	0.483	−0.602	0.063	1							
**Sand**	−0.588	−0.590	0.292	0.112	1						
**Silt**	0.533	0.131	0.291	0.300	−0.547	1					
**Clay**	0.052	0.501	−0.534	−0.456	−0.499	−0.447	1				
**PI**	−0.578	−0.254	0.093	−0.225	0.542	−0.556	0.013	1			
**MBV**	−0.540	−0.280	0.176	−0.183	0.221	−0.170	−0.036	0.428	1		
$\phi_{UU}$	0.884	0.688	−0.281	0.101	−0.751	0.602	0.157	−0.595	−0.547	1	
**CUU**	0.739	0.035	−0.174	0.558	−0.493	0.666	−0.201	−0.685	−0.637	0.635	1
	Strong negative correlations (<−0.7)										
	Moderate negative correlations (−0.7–(−0.4))										
	Negligible/weak negative correlations (−0.4–0)										
	Negligible/weak positive correlations (0–0.4)										
	Moderate positive correlations (0.4–0.7)										
	Strong positive correlations (>0.7)										

ρ_s_ and ρ_b_: particle and bulk densities (in g/cm^3^), W: water content (in wt.%), n: porosity (in %), PI: plasticity index (in %), MBV: methylene blue value (in g/100g), ϕ_UU_: friction angle (in °), C_UU_: cohesion (in kilo Pascal).

**Table 4:** Average soil geomechanical values with the corresponding soil types.

Soil Properties	Soil on Basalt	Soil on Trachy-Rhyolite	Soil on Migmatite	Rhyolite Outcrop
$\rho_{s}\left(g/cm^{3}\right)$	2.66	2.59	2.9	26.51
$\rho_{b}\;\left(g/cm^{3}\right)$	1.50	1.45	1.58	0
**W (wt%)**	41.2	44.1	39.3	0
**n (%)**	44	43	45	0
**Sand (%)**	49	53	31	0
**Silt (%)**	21	19	27	0
**Clay (%)**	29	28	42	0
**PI (%)**	27.6	33	15.9	0
**MBV (g/100g)**	2	1.6	1.5	0
$\phi_{UU}\;\left({}^{\circ}\right)$	23.6	16.8	31.5	46
**CUU (kPa)**	25.4	33.2	47.7	1000

ρ_s_ and ρ_b_: particle and bulk densities (in g/cm^3^), W: water content (in wt.%), n: porosity (in %), PI: plasticity index (in %), MBV: methylene blue value (in g/100g), ϕ_UU_: friction angle (in °), C_UU_: cohesion (in kilo Pascal)

**Table 5:** Geomechanical properties whose decreasing tendency makes the site more susceptible to landslides: particle and bulk densities, internal angle of friction, and cohesion.

Soil Properties.	Classes	Fuzzy Membership Values
Particle density	26.5	0
(g/cm^3^)	15.8	0.89
	15.3	0.94
	14.6	0.99
Bulk density	2.9	0
(g/cm^3^)	2.65	0.008
	2.62	0.009
	0	1
Cohesion	1000	0
(kPa)	47.7	0.97
	33.2	0.99
	25.39	1
Friction angle	46	0
(°)	31.5	0.69
	23.6	1
	16.79	1

**Table 6:** Soil geomechanical properties whose increasing behavior augments the probability of landslide: porosity, water content, methylene blue value (MBV), plasticity index (PI), sand, and clay content.

Soil Properties.	Classes	Fuzzy Membership Values
Porosity	0	0
(%)	41.4	0.9
	44.5	0.97
	45.38	1
Water content	0	0
(wt%)	39.11	0.92
	39.29	0.93
	44.32	1
MBV	0	0
(g/100 g)	1.28	0.83
	1.51	0.98
	1.53	1
PI	0	0
(%)	15.89	0
	27.39	0.82
	30.39	1
Sand content	0	0
(%)	31	0.43
	50.33	1
	52	1
Clay content	0	0
(%)	27	0.64
	29.33	0.69
	42	1

MBV: methylene blue value, PI: plasticity index.

**Table 7:** (**a**) Computed weights of geo-environmental factors classes using the information value method: slope (°), aspect (°), elevation (m), land cover, distance to river (m); (**b**) Computed weights of geo-environmental factors classes using the information value method: distance to roads (m), curvature (°), and lithology.

**(a)**				
**Factors**	**Class**	**Number of Pixels**	**Number of Landslide Pixels**	**Weight**
Slope	0–6	742,206	68	−1.39
	6–9	844,882	123	−1.19
	9–2	821,644	225	−0.91
	12–16	774,074	379	−0.66
	16–19	806,224	628	−0.46
	19–24	801,953	1154	−0.19
	24–31	768,216	2594	0.18
	>31	744,791	8896	0.73
Aspect	North (0–22.5)	396,461	1168	0.12
	Northeast (22.5–67.5)	702,971	1051	−0.17
	East (67.5–112.5)	643,290	316	−0.66
	Southeast (112.5–157.5)	743,728	201	−0.92
	South (157.5–202.5)	955,132	1081	−0.30
	Southwest (202.5–247.5)	889,292	2516	0.10
	West (247.5–292.5)	772,714	3367	0.29
	Northwest (292.5–337.5)	790,985	3012	0.23
	North (337.5–360)	409,417	1355	0.17
Elevation	807–1348 m	764,218	0	−3.00
	1348–1479 m	783,308	704	−0.52
	1479–1574 m	832,807	1828	−0.13
	1574–1731 m	818,594	4151	0.24
	1731–1880 m	766,924	3998	0.25
	1880–2036 m	798,062	1967	−0.08
	2036–2228 m	764,037	1080	−0.32
	>2228 m	776,040	339	−0.83
Land cover	Barren land	514,356	730	−0.20
	Shrub land	2,339,193	3828	−0.14
	Shrub with emergent trees	1,388,984	1840	−0.23
	Forest	2,061,457	7669	0.22
Distance to river	0–200 m	1,754,147	2777	−0.15
	200–400 m	1,454,202	2744	−0.07
	400–600 m	1,225,604	3755	0.14
	600–800 m	923,524	2724	0.12
	800–1000 m	551,280	1927	0.20
	1000–1200 m	249,712	140	−0.60
	1200–1400 m	84,213	644	0.54
	>1400 m	61,308	10,035	1.87
**(b)**				
**Factors**	**Class**	**Number of Pixels**	**Number of Landslide Pixels**	**Weight**
Distance to roads (m)	0–200 m	1,204,169	13	−2.32
	200–400 m	919,356	441	−0.67
	400–600 m	732,736	402	−0.61
	600–800 m	597,762	468	−0.45
	800–1000 m	496,625	816	−0.13
	1000–1200 m	413,751	1248	0.13
	1200–1400 m	347,459	644	−0.08
	>1400 m	1,590,881		0.45
Curvature (°)	−314–(−6)	188,169	2831	0.83
	−6–(−3)	352,288	1243	0.20
	−3–0	1,857,582	2324	−0.25
	0–2	2,975,545	2868	−0.36
	2–4	647,298	1626	0.05
	4–7	152,047	900	0.42
	7–13	82,842	981	0.73
	>13	48,219	1294	1.08
Lithology	Trachy-rhyolite	2,319,151	9632	0.27
	Rhyolite	2200	0	−3.00
	Migmatite	653,933	32	−1.67
	Basalt	3,327,529	4403	−0.23

**Table 8:** Characteristics of the receiver operator characteristic (ROC) fitting curves exponential equation.

Models	TPR_0_	A_1_	t_1_ (threshold)	R^2^
Geo-environment	1 (±0)	−0.64553 (±0.02502)	0.31735 (±0.02561)	0.82519
Geo-environment + soil properties	1 (±0)	−0.66248 (±0.01818)	0.09695 (±0.01716)	0.79462

TPR_0_: true positive rate, A_1_: coefficient, FPR: false positive rate, t_r_: threshold.
